# S100A11 Promotes Acute Pancreatitis by Upregulating Acinar Cell Ferroptosis

**DOI:** 10.1155/mi/6971024

**Published:** 2025-06-12

**Authors:** Huiyun Zhu, Hongxin Sun, Xianzhu Zhou, Ge Li, Yun Zhang, Youhan Zhang, Jia Gui, Siying Fei, Xiaoyang Dong, Xiaoju Su, Yan Chen, Cui Chen, Yiqi Du

**Affiliations:** ^1^Department of Gastroenterology, The First Affiliated Hospital of Naval Medical University, 168 Changhai Road, Shanghai 200433, China; ^2^Clinical Research Center, The First Affiliated Hospital of Naval Medical University, 168 Changhai Road, Shanghai 200433, China

**Keywords:** acute pancreatitis, ferroptosis, pancreatic acinar cells, S100A11

## Abstract

Acute pancreatitis (AP) is a common gastrointestinal disease that can cause systemic inflammation and lead to dysfunction of multiple organs. In pancreatitis, ferroptosis promotes disease progression and organ damage by regulating oxidative stress and inflammatory response. Here, ferroptosis was significantly elevated in the AP rat model and participated in regulating disease progression. Meanwhile, the expression of S100A11 was significantly upregulated in the pancreatic tissue of rats with AP, as determined by tandem mass spectrometry (TMT) proteomics. This phenomenon was also confirmed in pancreatic acinar cells. To reveal whether S100A11 participates in regulating ferroptosis, an S100A11 knockdown lentivirus was transfected into caerulein-treated pancreatic acinar cells AR42J. Functional results revealed that S100A11 knockdown significantly increased cell viability and GSH levels, while decreasing reactive oxygen species (ROS), lipid ROS, and Fe^2+^ levels in pancreatic acinar cells compared to the control group. In vivo, S100A11 knockdown via adeno-associated virus inhibited caerulein-induced ferroptosis. These findings suggest that S100A11 promotes AP by upregulating ferroptosis, which exacerbates oxidative stress and inflammation in pancreatic tissue.

## 1. Introduction

Acute pancreatitis (AP) is an inflammatory disease caused by abnormal activation of pancreatic enzymes, which may trigger a systemic inflammatory reaction and impair the function of multiple organs [1, 2]. Multiple injurious factors (gallstones, high triglycerides, alcohol, etc.) and other factors may damage pancreatic acinar cells and initiate early localized inflammation, leading to cell death [3]. Cell death results in pathologic systemic damage in severe AP due to activation of digestive enzymes and other inflammatory pathways [4, 5]. Therefore, targeted modulation of cell death plays a therapeutic role in the treatment of severe AP.

Oxidative stress plays a crucial role in the local and systemic inflammatory responses of AP [6]. Lipid peroxide modifications in the membrane bilayer are involved in the regulation of cell fate [7]. In the context of cellular oxidative stress, the massive accumulation of lipid peroxides leads to a novel mode of cell death with iron-dependent ferroptosis [7, 8]. When cellular conditions block the cystine/glutamate transporter (Xc-) system, glutathione (GSH) peroxidase-4 (GPX4) axis [9], ferroptosis often occurs, characterized by iron accumulation, intense lipid peroxidation, and reactive oxygen species (ROS) generation [10, 11]. The depletion of GSH induced by the system Xc- and subsequent decrease in GPX4 activity are the major factors inducing ferroptosis. System Xc- is composed of a disulfide-linked heterodimer between Xc transporter, which is encoded by the solute carrier family 7 member 11 (SLC7A11) gene, and the surface antigen heavy chain, encoded by the solute carrier family 3 member 2 (SLC3A2) gene. The system is crucial for the import of cysteine [12, 13]. According to reports, inhibition of cystine import depletes intracellular GSH levels, leading to inactivation of GPX4 activity and increases production of lipid ROS. GPX4 plays a crucial role in ferroptosis; its inactivation leads to lipid peroxidation, oxidative imbalance, membrane structural dysfunction, and abnormal iron metabolism [14]. The pathogenesis of AP involves various types of cell death. For instance, research has shown that in the process of AP, iron-dependent cell death exacerbates pancreatic damage and systemic inflammatory response [15]. Recent research progress shows that imbalances in iron metabolism and GPX4-mediated ferritin deposition play significant roles in the pathological progression of AP [16]. However, the detailed molecular mechanisms of ferroptosis in AP progression still need further exploration.

The S100 protein family consists of over 20 members involved in a wide range of intracellular and extracellular functional regulation [17]. S100A11, originally discovered in smooth muscle, regulates various biological functions such as enzyme activity, endocytosis, cell proliferation, transcriptional regulation, and cell differentiation [18, 19]. Recent studies have revealed a close association between S100A11 and inflammation [20, 21]. Previous research has also shown that S100A11 is associated with low-level inflammation in osteoarthritis (OA) [22]. In the synovial fluid of rheumatoid arthritis (RA) patients, S100A11 accumulates extensively and correlates with inflammation and disease progression [23]. Studies have shown that the regulation of S100A11 expression is associated with tumor metastasis [24–26]. Studies have shown that S100A11 accelerates the progression of pancreatic cancer by regulating various malignant biological behaviors [27–29]. However, the effect of S100A11 on AP remains unreported, necessitating further investigation.

In this research, we successfully constructed an animal model of AP with caerulein. Based on tandem mass spectrometry (TMT) proteomics, we found that S100A11 was significantly upregulated in AP tissues, and this result was also confirmed in AP cell models. Cell and animal experiments showed that S100A11 was involved in the ferroptosis of pancreatic acinar cells induced by caerulein. Our research provides a new insight into the pathogenesis of AP.

## 2. Materials and Methods

### 2.1. Cell and Animal Models

Rat pancreatic exocrine cells (AR42J) were obtained from Cyagen Biosciences (Shanghai) Inc. and cultured in F-12K medium with 10% fetal bovine serum (Hyclone, Logan, UT, USA) at 37°C in a 5% carbon dioxide (CO_2_) environment under normal oxygen conditions. AR42J cells treated with 10 nM caerulein were used as a cell-culture model of AP. Twelve adult male Sprague–Dawley (SD) rats were purchased from SHANGHAI MEDEL ORGANISMS (Shanghai, China) and raised in an SPF environment. They were maintained on a 12-hour light–dark cycle with access to both food and drinking water. The rat model of AP was established by intraperitoneal injection of caerulein dissolved in PBS (50 µg/kg/dose), administered once every 1 h for 4 times. The control group was injected with an equal volume of PBS. Rats were euthanized by asphyxiation with excess carbon dioxide. To investigate the effect of ferroptosis on AP, rats were treated with the ferroptosis inhibitor Ferrostatin-1 (Fer-1, 10 mg/kg, i.p.) 1 h before model establishment, and the disease characteristics of AP were observed. To achieve S100A11 knockdown in mouse pancreatic tissue, we injected AAV virus serotypes 8 (1 x 10^11^ vg/per mouse) through the pancreatic duct and conducted AP modeling by caerulein 1 month after injection. Six animals were used in each group.

### 2.2. Histology Staining

The pancreas was excised and fixed with 4% paraformaldehyde for 48 h. The pancreas tissues were cut into 5 μm-thick sections after paraffin embedding. These prepared sections were stained with hematoxylin–eosin (H&E). In brief, for H&E analysis, pancreatic tissue sections were dewaxed with xylene and progressively dehydrated with ethanol, stained with hematoxylin, then decolorized with tap water and ethanol hydrochloride, and then stained with eosin. For the quantification of H&E staining, the staining intensity score was defined based on three criteria: inflammatory cell infiltration (score 1–3), erythrocyte hemorrhage (score 1–3), and islet morphology (score 1–3). The film was sealed with neutral resin and photographed under a microscope. Microscopical photographs were taken with an Olympus camera (Tokyo, Japan).

### 2.3. TMT Proteomics and Bioinformatic Analysis

Proteins were lysed using SDT buffer and digested using the filtration-assisted sample preparation (FASP) method. Peptides were desalted, concentrated, and reconstituted in 0.1% formic acid. The 100 μg peptide mixture of each sample was labeled TMT, and then LC-MS/MS analysis was performed on a Q Exactive mass spectrometer connected to Easy nLC, operating in positive ion mode. In addition, for the screening of significantly different proteins, the expression rate is defined as fold change (FC), with FC greater than 1.2 or less than 0.83 and *p* -value < 0.05 as the criteria for upregulated or downregulated expression. Afterwards, the raw MS data were used for homologous sequence alignment and analyzed using NCBI-BLAST and InterProScan. Subsequently, the R script was used to annotate the GO and KEGG functions of the sequences.

### 2.4. Real-Time Quantitative Reverse Transcriptase Polymerase Chain Reaction (qRT-PCR)

Total RNA from 1x105 cells was obtained using RNAiso Plus (9109, Takara). Complementary DNA (cDNA) reverse transcription was performed using the FastKing cDNA First-Strand Synthesis Kit (KR116, Tiangen). Then, qRT-PCR was detected by SYBR Premix Ex Taq polymerase (TaKaRa Biotechnology (Dalian) Co., Ltd., Dalian, China). Glyceraldehyde 3-phosphate dehydrogenase (GAPDH), flg2, mgst1, and s100a11 were amplified with specific primers, as shown in Table 1.

### 2.5. Western Blot Analysis

Total cell protein lysates were prepared in radioimmunoprecipitation assay buffer. Proteins were separated using standard sodium dodecyl sulfate polyacrylamide gel electrophoresis and transferred to polyvinyl difluoride membranes (IPVH00010, Merck-Millipore), which were washed, blocked, and probed with specific primary antibodies against S100A11 (Abcam, Cambridge, MA, USA), Mgst1 (Abcam), Fgl2 (Abcam), FTH1 (Abcam), GPX4 (Abcam), ACSL4 (Abcam), or GAPDH (Abcam), followed by horseradish peroxidase-conjugated secondary antibodies. Finally, protein bands were detected and quantified. Relative protein expression was all normalized to that of GAPDH.

### 2.6. The shRNA-Mediated S100A11 Gene Knockdown

Human S100A11 shRNA was obtained from a GIPZ lentiviral shRNA library (GE Dharmacon, Lafayette, CO, USA). HEK-293FT cells were co-transfected with psPAX2 and PMD2.G packaging plasmids coding for S100A11 shRNA (shRNA-1^#^: GATTTCCAAGAGTTTCTCAACCTTA, shRNA-2^#^: AGAAGTATAGTGGGAAGGATGGAAA, shRNA-3^#^: GCATGATGAAGAAGCTGGACCTCAA) and a negative control shRNA using Lipofectamine 3000 reagent (Life Technologies, Carlsbad, CA, USA). The lentiviral particles were transfected into pancreatic acinar cells AR42J with polybrene, and then the cells successfully infected with the lentivirus were screened positively with 2 µg/mL puromycin.

### 2.7. Cell Growth Assay

After infection with either sh-S100A11 or sh-ctrl lentivirus, the infected AR42J cells were treated with the above cultured condition. Briefly, 10 µl CCK-8 solution was added to each well and incubated under the same conditions for 4 h. The absorbance, directly correlating with cell proliferation, was detected at 450 nm using a spectrophotometer.

### 2.8. Cellular ROS Detection

The intracellular ROS level was measured using the oxidative-sensitive fluorescent probe DCFH-DA. Simply put, after infecting the cells with either sh-S100A11 or sh-ctrl lentivirus, the cells were cultured, washed, and then incubated with 5 µM DCFH-DA at 37°C in the dark for 30 min. Then, the DCFH fluorescence signal, directly correlating with ROS level, was detected at 488 nm using a microplate reader.

### 2.9. GSH Quantification, Lipid Peroxidation, Fe^2+^ Detection

The GSH content in cells or tissues was quantified using a GSH assay kit (S0053, Beyotime). The Fe^2+^ level was measured with a ferrous ion colorimetric assay kit (BC5410, Solarbio). Lipid peroxidation was estimated using the BODIPY 581/591 C11 kit (S0043, Beyotime) and calculated based on the red/green fluorescence ratio, cells or tissues detection.

### 2.10. Transmission Electron Microscopy

Cells were fixed with 1 mL of 2.5% glutaraldehyde and placed at 4°C for 4–6 h. Then, the cells were washed, dehydrated, embedded, and sectioned into 65 nm ultrathin sections. The sections were stained with 1% uranyl acetate and 0.1% lead citrate for 10 min each. After washing, the sections were observed using a transmission electron microscope (JEOL, Pleasanton, California, USA).

### 2.11. Statistical Analysis

In statistical analysis, the statistical data were analyzed with GraphPad Prism 8 software and presented as the mean ± SD. The significance data between the two groups was evaluated using *t*-test. Three or more groups of data were analyzed using one-way ANOVA, with Tukey's post hoc test. The significance level of the results is usually expressed as a *p*-value, and less than 0.05 is considered statistically significant. Significance level through the symbol *⁣*^*∗*^, *⁣*^*∗∗*^, *⁣*^*∗∗∗*^/*⁣*^*∗∗∗∗*^ to indicate the different levels of significance, respectively corresponding to *p* < 0.05, 0.01, 0.001.

## 3. Results

### 3.1. Ferroptosis Was Involved in Caerulein-Induced AP

**Figure 1:**
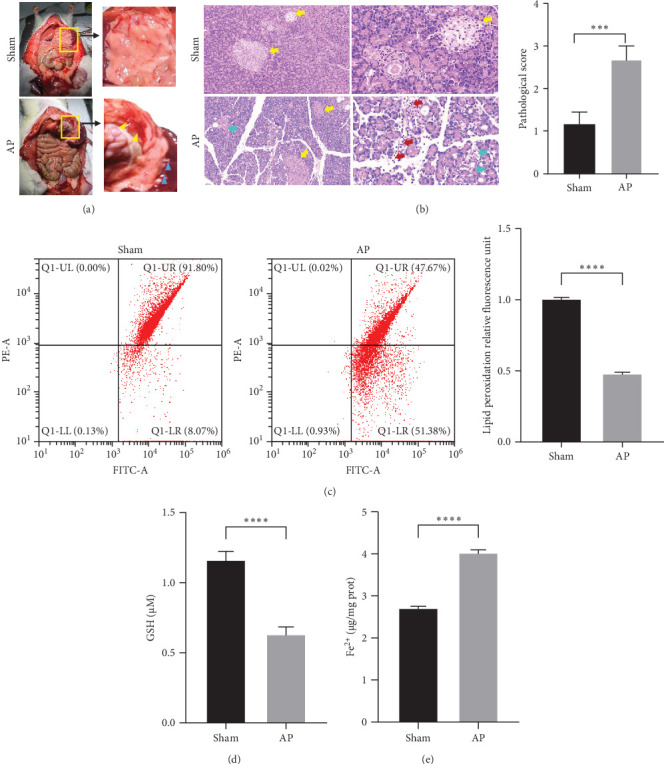
Morphological and histological analysis of acute pancreatitis. (A) Gross anatomic of acute pancreatitis. (B) H&E staining was used to analyze the histopathology at different magnification (x100 and x200), and scored based on histology; yellow arrow: pancreatic tissue structure, blue arrow: red blood cell, and red arrow: neutrophils. (C) Lipid ROS levels in sham and AP rats were measured using the BODIPY 581/591 C11 assay and quantified based on the relative fluorescence ratio of lipid peroxidation (red/green). (D) GSH levels in sham and AP rats were measured using a GSH assay kit. (E) Fe^2+^ levels in sham and AP rats were measured using a ferrous ion colorimetric assay kit. *⁣*^*∗∗∗*^*p* < 0.001, *⁣*^*∗∗∗∗*^*p* < 0.0001.

**Figure 2:**
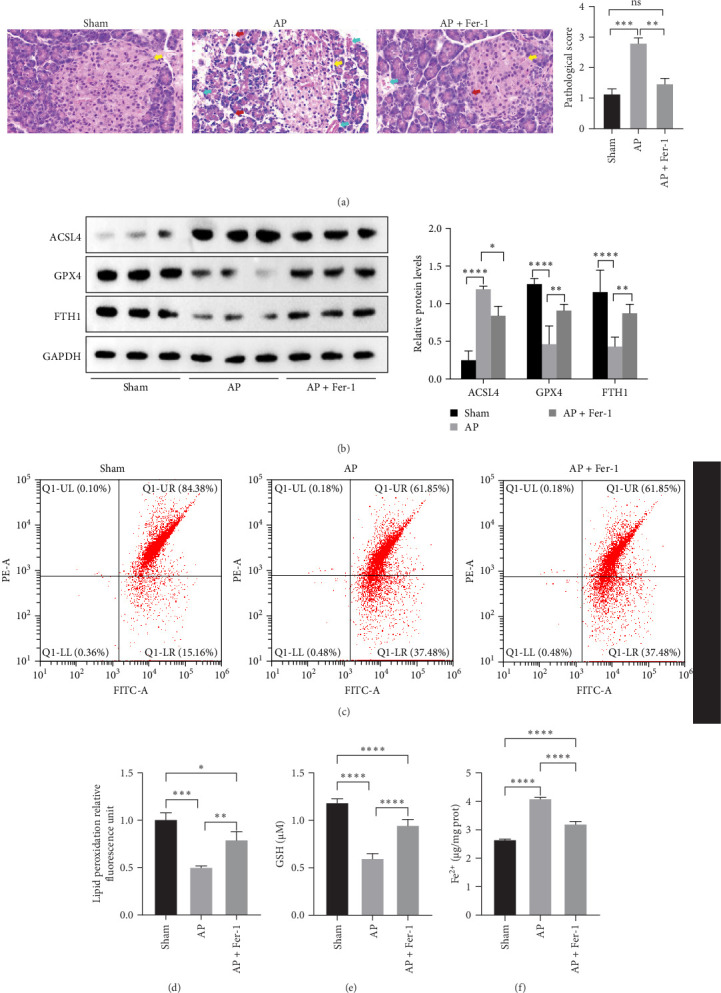
Ferrostatin-1 (Fer-1) treatment alleviates caerulein-induced AP. (A) H&E staining was performed at different magnification (x63) to assess histopathological changes, and followed by histological scoring; yellow arrow: pancreatic tissue structure, blue arrow: red blood cell, red arrow: neutrophils. (B) Western blotting was used to detect and quantify the expression levels of ferroptosis-related proteins (ACSL4, GPX4, and FTH1) in the pancreatic tissue from sham and AP rats. (C) Lipid ROS levels in sham and AP rats were measured using the BODIPY 581/591 C11 assay. (D) Lipid ROS levels were quantified based on the quantified based on the relative fluorescence ratio of lipid peroxidation (red/green). (E) GSH levels in sham and AP rats were measured using a GSH assay kit. (F) Fe^2^⁺ levels in sham and AP rats were measured using a ferrous ion colorimetric assay kit. ns, not significant,*⁣*^*∗*^*p* < 0.05, *⁣*^*∗∗*^*p* < 0.01, *⁣*^*∗∗∗*^*p* < 0.001, *⁣*^*∗∗∗∗*^*p* < 0.0001.

To investigate the pathogenesis of AP, SD rats were used to construct an animal model by intraperitoneal injection of caerulein, and pancreatic tissue samples were collected after 24 h. Histological evaluation demonstrated that compared with the control group, the pancreatic tissue of rats in the model group showed pancreatic fluid exudation, capillary rupture and hemorrhage (yellow arrow), and tissue adhesion (blue arrow), indicating that the model was successfully constructed (Figure 1A). The pancreatic tissue was partially fixed with paraformaldehyde for pathological detection, and partially frozen with liquid nitrogen in a −80°C refrigerator for molecular detection and proteomics. H&E results showed that the pancreatic tissue structure was intact, the acini were closely arranged, and the islets were visible (yellow arrow) in the control group. In contrast, the model group exhibited disrupted pancreatic tissue architecture, the acinar boundaries were blurred, red blood cells were scattered between the acinar cells (blue arrow), and a large number of neutrophils (red arrow) infiltrated the interstitial cells characterized by acute inflammation, indicating the existence of diffuse bleeding in the pancreatic tissue (Figure 1B). Next, we quantified lipid peroxidation in the pancreatic tissue of rats, a hallmark index of ferroptosis. The results revealed a notably decreased red/green ratio in the AP model, indicating an elevated lipid ROS level (Figure 1C). Additionally, compared with the sham group, GSH level was notably reduced (Figure 1D) and the Fe^2+^ content was markedly increased (Figure 1E) in the AP model. Furthermore, we treated AP rats with the ferroptosis inhibitor Fer-1 and observed that Fer-1 treatment effectively suppressed the progression of caerulein-induced AP, as evidenced by H&E staining. The results showed that Fer-1 treatment reduced pancreatic tissue disorganization, erythrocyte distribution, and neutrophil infiltration in the AP group (Figure 2A). Moreover, Fer-1 treatment markedly downregulated ACSL4 expression while upregulating GPX4 and FTH1 expression (Figure 2B), indicating a shift towards ferroptosis inhibition. Additionally, Fer-1 significantly reduced lipid peroxidation levels (Figure 2C,D) and Fe^2+^ concentrations (Figure 2F), while increasing GSH levels (Figure 2E). Collectively, these findings suggest that Fer-1 effectively attenuates ferroptosis and mitigates pancreatic injury, highlighting its potential protective role in the progression of AP.

### 3.2. TMT Proteomics Was Used to Screen Differentially Expressed Proteins

**Figure 3:**
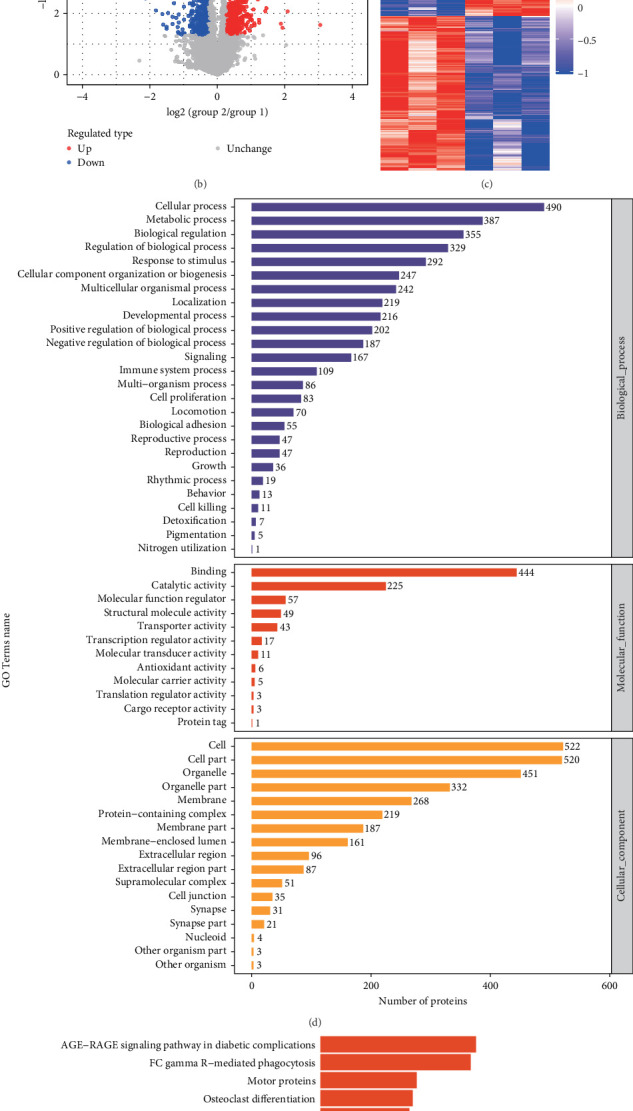
Differentially expressed proteins were screened by TMT. (A) Statistical results of the number of upregulated and downregulated proteins in the model group compared to the control group. (B) The volcano map showed differential proteins. (C) Cluster analysis of differential genes. (D) GO function and (E) KEGG pathway were analysis by bioinformatics analysis.

TMT-based proteomics was employed to identify differentially expressed proteins in pancreatic tissue between control and model groups. The results indicated 460 proteins were significantly upregulated and 416 proteins were downregulated in the model group (Figure 3A). These differentially expressed proteins were visualized in a volcanic plot (Figure 3B). To analyze the expression patterns between the control and model groups and validate the grouping rationale of this project, the differentially expressed proteins were clustered and visualized as a heatmap. A screening criterion of fold change >1.2 or <0.83 and *p*-value < 0.05 was used, effectively distinguished the two groups based on significantly differentially expressed proteins (Figure 3C). Further, Blast2Go software was utilized to annotate the GO functions of differentially expressed proteins. The “cellular process” terms of biological process, “binding” terms of molecular function, and “cell” and “cell part” terms of cell component were remarkably involved (Figure 3D). In addition, the differentially expressed proteins were annotated using the Kyoto Encyclopedia of Genes and Genomes (KEGG) pathway database (Figure 3E).

### 3.3. Caerulein Promotes Ferroptosis In Vivo and In Vitro, With S100A11 Increasing

**Figure 4:**
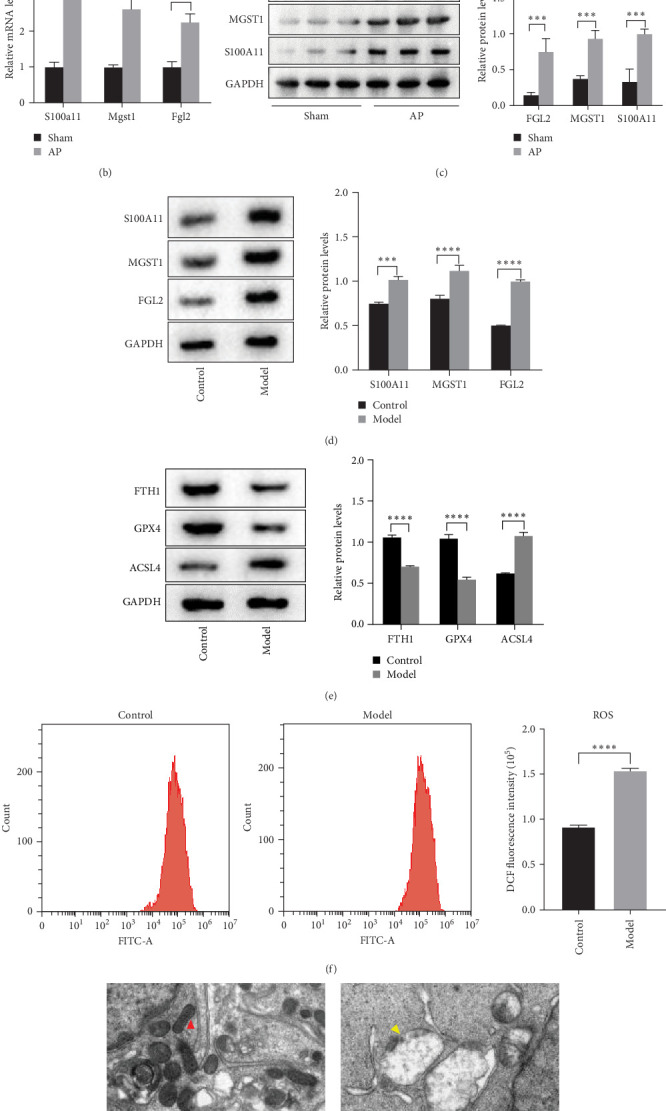
Caerulein promotes ferroptosis in vivo and in vitro with increased S100A11 expression. (A) Proteins identified through differential expression analysis. (B) The mRNA expression levels of Fgl2, Mgst1, and S100A11 in tissues from sham and AP models were analyzed by qPCR. (C) The protein expression of Fgl2, Mgst1, and S100A11 in tissues from sham and AP models was confirmed and quantified by western blot and ImageJ software. (D) The protein expression levels of S100A11, Fgl2, and Mgst1 in cells with or without caerulein treatment were detected by western blot and quantified using ImageJ software. (E) The expression levels of ferroptosis-related proteins (FTH1, GPX4, and ACSL4) were analyzed and quantified by western blot and ImageJ software. (F) The DCFH-DA assay was used to measure ROS levels. (G) Electron microscopy was used to analyze the mitochondrial morphology in AR42J cells. *⁣*^*∗∗∗*^*p* < 0.01, *⁣*^*∗∗∗∗*^*p* < 0.0001.

Based on the differential protein expression list, we identified three significantly expressed proteins (S100A11, MGST1, FGL2) (Figure 4A). Both q-PCR and western blotting results further showed increased expression of S100A11, MGST1, and FGL2 in line with the tissue results (Figure 4B,C).

To further validate the expression of these proteins, pancreatic acinar cells AR42J were treated with caerulein to establish a cell model of AP. Western blotting confirmed the increased expression of S100A11, MGST1, and FGL2 in the cell model group (Figure 4D). Meanwhile, ferroptosis-related proteins ACSL4 were increased, while FTH1 and GPX4 were decreased in the cell model group (Figure 4E). The results of the ROS detection kit showed that ROS was increased in the cell model group (Figure 4F). Electron microscopy showed that mitochondria o in the control group pancreatic acinar cells exhibited regular, elliptical, and rod-shaped structures, surrounded by a double-layer membrane. The inner membrane protrudes inward into a plane-like ridge, and the ridge is vertically aligned with the long axis of mitochondria (red arrow). In contrast, mitochondria were significantly reduced in number, exhibited abnormal morphology with prominent vacuolization, blurred ridges, and a pale appearance in the model group (yellow arrow) (Figure 4G). These findings indicated that S100A11 was upregulated in both tissue and cell samples of the model group. Furthermore, the occurrence of ferroptosis was dramatically increased in response to caerulein treatment.

### 3.4. S100A11 Knockdown Inhibits Ferroptosis Induced by Caerulein In Vitro

**Figure 5:**
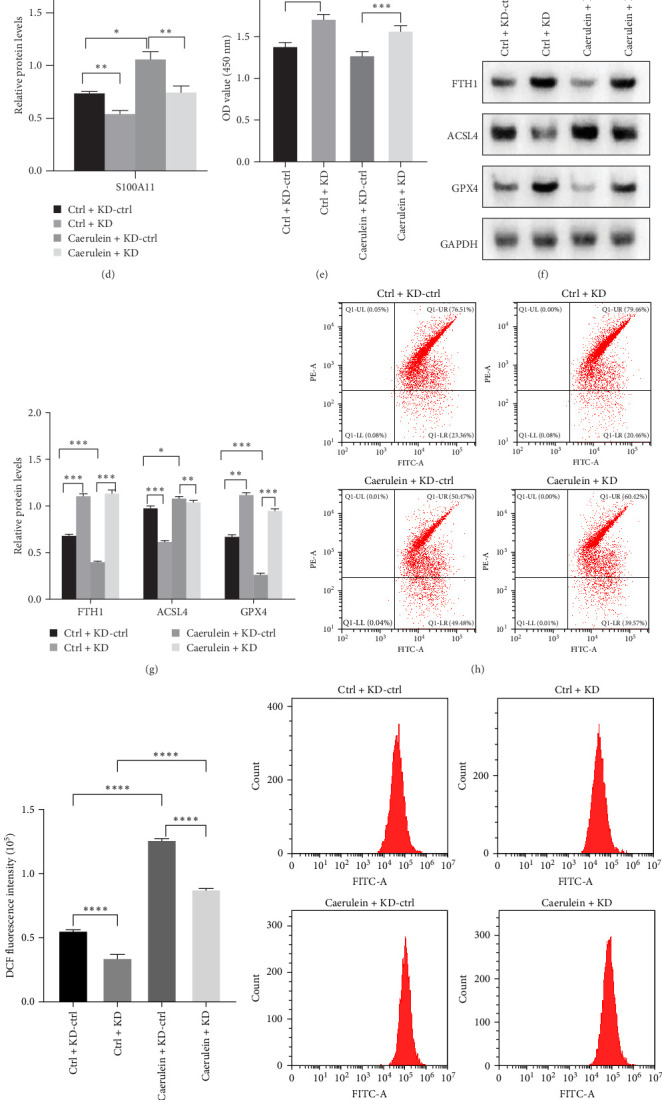
S100A11 knockdown inhibits caerulein-induced ferroptosis in vitro. (A) AR42J cells were infected with shS100A11 lentivirus targeting three different sites, and S100A11 mRNA levels were analyzed by qPCR. (B) S100A11 mRNA levels were analyzed by qPCR in AR42J cells with or without caerulein treatment. (C, D) S100A11 expression was analyzed and quantified in shS100A11-infected AR42J cells with or without caerulein treatment. (E) CCK-8 assay was used to assess AR42J cell proliferation with or without caerulein treatment. (F, G) The protein levels of ferroptosis-related proteins (FTH, GPX4, and ACSL4) were analyzed and quantified in AR42J cells with or without caerulein treatment. (H, I) Lipid ROS levels in AR42J cells with or without caerulein treatment were measured using the BODIPY 581/591 C11 assay and then quantified based on the relative fluorescence ratio of lipid peroxidation (red/green). (J, K) Intracellular ROS levels were measured using DCFH-DA staining. (L) GSH levels in AR42J cells with or without caerulein treatment were measured using a GSH assay kit. (M) Fe^2^⁺ levels in AR42J cells with or without caerulein treatment were measured using a ferrous ion colorimetric assay kit. ns, not significant; *⁣*^*∗*^*p* < 0.05; *⁣*^*∗∗*^*p* < 0.01; *⁣*^*∗∗∗*^*p* < 0.001; *⁣*^*∗∗∗∗*^*p* < 0.0001.

To further investigate the role of S100A11 in caerulein-induced ferroptosis, a specific S100A11 knockdown lentivirus was constructed and transfected into pancreatic acinar cells AR42J. The expression efficiency was verified by qPCR, confirming a significant decrease in S100A11 expression (Figure 5A). Moreover, we established an AR42J cell line infected with S100A11 knockdown lentivirus, with or without caerulein treatment. The results showed that S100A11 knockdown reduced its expression in both the control and model groups of AR42J cells (Figure 5B-D). Cell viability was assessed using a cell counting kit, and the results showed whether treated with caerulein, compared with the control group, S100A11 knockdown promotes cell viability. However, S100A11 knockdown combined with caerulein treatment inhibited cell viability compared to S100A11 knockdown alone (Figure 5E). Analysis of ferroptosis-related protein expression revealed that ACSL4 levels were decreased, while FTH1 and GPX4 levels were increased, indicating reduced ferroptosis with S100A11 knockdown (Figure 5F,G). Flow cytometry revealed that S100A11 knockdown inhibited ROS production in both caerulein-treated and untreated cells compared with the control group (Figure 5H,I). Additionally, we observed that S100A11 knockdown reduced lipid peroxidation levels (Figure 5J,K), increased the antioxidant GSH level (Figure 5L), and elevated Fe^2⁺^ levels (Figure 5M) in both the control and AP model AR42J cells, indicating a significant inhibitory effect on ferroptosis.

### 3.5. S100A11 Knockdown Inhibits Ferroptosis Induced by Caerulein In Vivo

**Figure 6:**
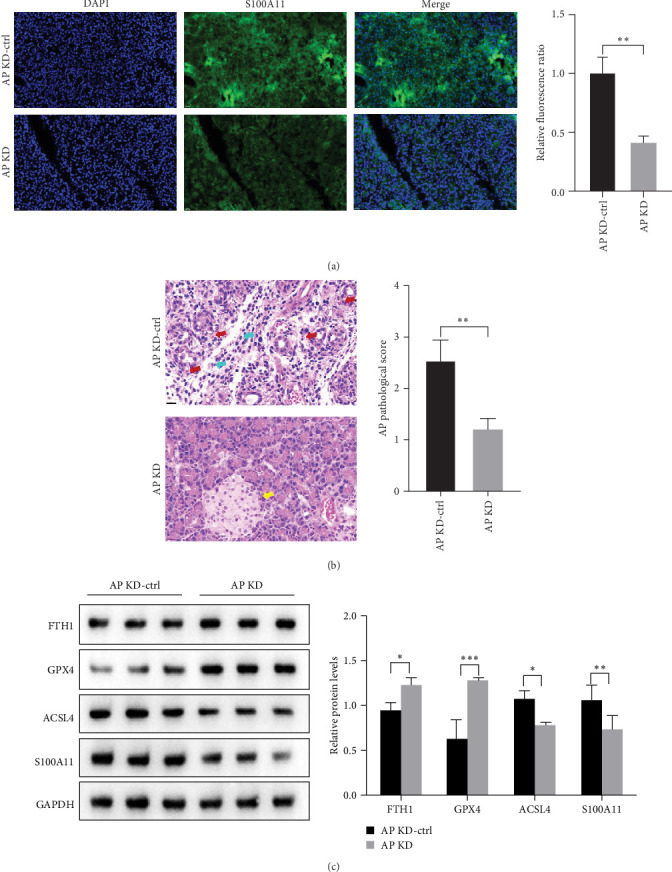
S100A11 knockdown inhibits caerulein-induced ferroptosis in vivo. (A) The knockdown efficiency of S100A11 in pancreatic tissue was analyzed by immunofluorescence staining. (B) H&E staining (x63) was used to analyze the histopathology of pancreatic tissue in KD-Ctrl and KD group, and scored based on histology; yellow arrow: pancreatic tissue structure, blue arrow: red blood cell, and red arrow: neutrophils. (C) The expression levels of ferroptosis-related proteins FTH1, GPX4, and ACSL4 were detected and quantified in KD-ctrl and KD group. *⁣*^*∗*^*p* < 0.05, *⁣*^*∗∗*^*p* < 0.01, *⁣*^*∗∗∗*^*p* < 0.001.

An animal experiment was used to verify the function of S100A11. Adeno-associated virus serotypes 8 were constructed and injected into the pancreatic ductal. 1 month later, the AP model was established by caerulein. Immunofluorescence results showed that the S100A11 knockdown was successful in AP (Figure 6A). H&E results showed that in the knockdown control (KD-ctrl) group, pancreatic tissue was disorganized, with blurred acinar boundaries and red blood cells (blue arrow). A large number of acute inflammatory neutrophils (red arrow) infiltrated the interstitium, indicating diffuse bleeding and acute inflammation in the pancreatic tissue (Figure 6B). In contrast, in the KD group, the pancreatic tissue structure was relatively complete, the acini were closely arranged, and the islets were visible (yellow arrow) (Figure 6B). Ferroptosis-related proteins were detected by western blotting. The result showed ACSL4 was decreased and FTH1 and GPX4 levels were increased in the KD group compared with the KD-ctrl group, which indicated S100A11 knockdown inhibits caerulein-induced ferroptosis (Figure 6C).

## 4. Discussion

In the present study, SD rats were used to establish an animal model of AP via intraperitoneal injection of caerulein, confirmed by H&E staining. Western blotting analysis of ferroptosis-related proteins indicated elevated ferroptosis in AP, consistent with previous research [30–32]. However, the exact mechanisms governing ferroptosis in AP are not fully understood. To address this, TMT proteomics was used to analyze pancreatic tissue in control and model groups. Differentially expressed proteins, GO terms, and KEGG pathways were analyzed. Notably, a key ferroptosis-related protein S100A11, was increased in the AP model and validated by q-PCR and western blot assays. Moreover, we investigated, for the first time, the relationship between S100A11 levels and ferroptosis in caerulein-induced AP, both in vitro and in vivo. These investigations suggested that reducing S100A11 levels inhibited ferroptosis triggered by caerulein. Our findings offer novel perspectives on the involvement of S100A11 in AP.

The exact pathophysiological mechanism of AP remains elusive [33]. However, significant progress has been made in understanding its basic processes over the past decade. Recently, various forms of regulatory cell death (RCD), such as apoptosis, ferroptosis, autophagy, necroptosis, etc., have emerged as crucial players in AP pathogenesis [2]. The progression of AP is closely related to the dynamic regulation and interaction of these different RCD pathways. Ferroptosis, a relatively recent form of cell death, is characterized by an overwhelming accumulation of lipid ROS and Fe^2+^, along with impaired, and mitochondrial function. In recent years, ferroptosis has been recognized as being closely associated with the pathogenesis of AP [15]. Research has shown that inhibiting ferroptosis can reduce ROS production and inflammation in AP, which may also have potential therapeutic benefits in preventing multiorgan failure [34]. Ferroptosis was created by Dr. Brent R. Stockwell's laboratory in 2012 and describes a novel regulated cell death mechanism. In instances of ferroptosis, cells exhibit necrosis-like changes characterized by membrane integrity loss, organelle swelling, and moderate chromatin aggregation [8]. Similar to the above studies, the AP model in this study exhibited characteristic features of ferroptosis, including elevated levels of ROS, lipid-ROS, and Fe^2+^ levels, as well as the abnormal expression of ferroptosis-related proteins and mitochondrial structure damage. These findings provide strong evidence for the successful establishment of the AP model and its close association with ferroptosis. Furthermore, the protective effect of the ferroptosis inhibitor Fer-1 was confirmed in the in vivo caerulein-induced AP model, as demonstrated by a significant reduction in lipid-ROS and Fe^2^⁺ levels, along with notable improvements in pancreatic histopathology. These results highlight the potential therapeutic role of Fer-1 in mitigating ferroptosis-related damage in AP.

Previously, studies have shown that an excess of ROS promotes inflammatory responses, which is an important factor in exacerbating AP [35–37]. The staining results of this study confirmed that S100A11 knockdown reduced ROS production in AP, reduced neutrophil recruitment by oxidative stress, and delayed pancreatic tissue ferroptosis. Therefore, S100A11 appears to protect pancreatic cells from ferroptosis in an oxidative stress-dependent manner.

Ferroptosis can be triggered via internal and external mechanisms, making GPX4 a potential target for either promoting or suppressing ferroptosis during the progression of AP [38, 39]. It has been reported that reduced GPX4 levels in mice can accelerate the development of experimental pancreatitis [40]. Wedelolactone has been shown to alleviate AP and associated lung injury via GPX4-mediated suppression of pyroptosis and ferroptosis [16]. In addition, studies have shown that FTH1 is responsible for maintaining the balance and storage of iron ions within cells, while ACSL4 mediates the binding of fatty acids to iron ions on the cell membrane, and abnormal expression of these genes can lead to the destruction of the cell membrane and eventual ferroptosis [41, 42]. A previous study has demonstrated that recombinant SQSTM1 protein inhibits acinar cell ferroptosis in AP by increasing the expression of ACSL4 and decreasing the expression of FTH1. This finding suggests that SQSTM1 may be used as a targeted molecule in the treatment of AP to interfere with the ferroptosis process by regulating the expression of ACSL4 and FTH1 [30]. In line with these findings, our current study found that S100A11 knockdown administration resulted in increased expression of FTH1 and GPX4 and decreased levels of ACSL4 in the pancreatic tissue of AP model rats. It is suggested that the knockdown of S100A11 might exert a protective effect against AP by suppressing ferroptosis.

The current study showed that S100A11 knockdown also effectively inhibited ferroptosis of pancreatic cells induced by AP, further confirming the significant role of S100A11 in the process of AP. However, this study has several limitations. First, the lack of an isotype control group to validate the specificity of S100A11 limits the interpretation of its specific role. Future studies should include additional control groups to further confirm its specificity. Second, this study primarily focused on exploring the role of S100A11 in AP through acinar cell functional experiments and animal studies, without extending to clinical research. Future studies will further explore its potential clinical significance, as well as its function in different etiologies of AP, chronic pancreatitis, other digestive system diseases, and systemic inflammatory diseases. Third, given the complex mechanisms underlying pancreatitis, additional molecular studies are needed to explore whether S100A11 directly interacts with genes involved in ferroptosis and to elucidate the specific mechanisms through which this interaction occurs. In summary, further research will be essential to fully understand its potential as both a biomarker and a therapeutic target.

## 5. Conclusion

In conclusion, administration of caerulein results in detrimental damage to pancreatic tissue in rats, triggering ferroptosis, ultimately culminating in AP. Notably, S100A11 knockdown by adeno-associated virus markedly reduced caerulein-induced ferroptosis in pancreatic tissue, suggesting that S100A11 could be used as a target for the prevention of AP.

## Figures and Tables

**Table 1:** Primers targeting the selected genes were used in RT-qPCR.

Gene	Primer sequences	Product length (bp)
*GAPDH*	F: 5′-ACGGCAAGTTCAACGGCACAG-3′	129
	R: 5′-CGACATACTCAGCACCAGCATCAC-3′	
*Fgl2*	F: 5′-CGACATTACAACCACGACCTGAC-3′	85
	R: 5′-AATAGAGCCCACAGTTCCCAGAG-3′	
*Mgst1*	F: 5′-CATCGTTCCCTTTCTCGGTATCG-3′	124
	R: 5′-AGTCAAGTAAGCAATGGTGTGGTAG-3′	
*S100a11*	F: 5′-CCTCGACCGCATGATGAAGAAG-3′	87
	R: 5′-GCTAAGCCGCCAATAAGGTTGAG-3′	

## Data Availability

The data that support the findings of this study are available upon request from the corresponding author. The data are not publicly available due to privacy or ethical restrictions.
